# Developing Professionalism in Dentistry: A Systematic Review

**DOI:** 10.15694/mep.2017.000085

**Published:** 2017-05-17

**Authors:** Tan Minh Nguyen, Derek Jones, Kinh Luan Ngo, Melanie Jane Hayes

**Affiliations:** 1 Deakin University; 2 The University of Edinburgh; 3 St Vincent's Dental Centre; 4 The University of Melbourne

**Keywords:** ethics, clinical education, professionalism, dental students, oral health therapy students

## Abstract

This article was migrated. The article was marked as recommended.

**Background:** Professionalism is a core competency and concern in all health professional education. Evidence from nursing and medicine suggests the evidence base for approaches to developing and assessing professionalism at undergraduate level is weak. In 2015, notifications, imposed sanctions, and in some cases de-registration against dental practitioners for reported incidences of breaches in infection control in New South Wales, Australia, have refreshed the essential need for dental practitioners to promote public safety and protection.

***Aim*:** To investigate the evidence for clinical education practice approaches to develop professionalism in dentistry.

**Methods:** Relevant electronic databases were searched for full-text peer reviewed papers relating to dental practitioners published between 2000 and June 2016 in English. All research designs were included. Following initial and detailed screening, included papers were independently quality appraised and strength of evidence graded by two independent reviewers.

**Results:** Removal of duplicates resulted in 195 unique papers; following screening 34 full text articles were assessed for eligibility resulting in 15 papers evaluated in this review. Eight different clinical education approaches were identified. Most studies were of low quality and reported low levels of educational outcomes based on Kirkpatrick’s Hierarchy. There is a lack of good quality evidence to support any one approach to develop professionalism in dentistry. What evidence there is focuses on low level educational outcomes such as learners experience.

**Conclusions:** Low level outcomes is common in educational research and therefore unsurprising. More disappointing was failure to adequately justify the methodology and the absence on the definition of dental professionalism. The research findings is consistent with the evidence across other health professions internationally. There is scope for an inter-professional approach to tackle the challenge developing and subsequently assessing professionalism.

## Introduction

Oral Health Therapy (OHT) is a relatively new established health profession, which evolved from the emerging needs of the community. The global patterns of oral disease, particularly dental caries (tooth decay), since the introduction of preventive measures like water fluoridation have resulted in less complex dental needs of the population towards self-care and minimal simple interventions ( Baltutis and Morgan, 1998). These less complex dental procedures can be provided by an oral health therapist, a dental practitioner who complements the role of the dentist. The profession integrates the scope of practice performed by dental therapists and dental hygienists, with education and training in oral health promotion. They are allied health professionals who provide a range of dental services. This includes comprehensive oral examinations, diagnostic, preventive, and surgical approaches of dental treatment. Any dental treatment needs of the client that are beyond the OHT scope of practice in the dentistry are referred to dentists and dental specialists.

At the Melbourne Dental School, there have been anecdotal personal stories shared by OHT students that they do not feel empowered to challenge unsupported evidence-based dental practice during external workplace-based clinical practice placements. Failure to practice (or question practice) that is not evidence-based is indicative of weaknesses in professionalism competency. This research was inspired to promote professionalism among OHT students on the merit that they may be appropriately placed to practice social justice.

## Literature Review

In Australia, professionalism is referred to as competencies and attributes of personal values, attitudes and behaviours by the accrediting institution for dental course programs (Australian Dental Council) and the health practitioner registration authority (Dental Board of Australia). The Dental Hygienists’ Association of Australia and the Australian Dental and Oral Health Therapists’ Association describes professionalism in their *Code of Ethics.* Across these organisations, professionalism in dentistry was not defined.

A definition of professionalism in dentistry was sought from the American and United Kingdom context. Likewise, a scoping search yielded no definition of professionalism in dentistry. Thus, the definition of professionalism was sought from the medical context. The American Board of Internal Medicine (ABIM) and the European Federation of Internal Medicine (EFIM) identified three fundamental principles of medical professionalism ( ABIM Foundation et al, 2005):

• Principle of primacy of patient welfare
• Principle of patient autonomy
• Principle of social justice

Based on the literature, the following definition of dental professionalism is proposed:

‘A set of competencies that incorporates the values, attributes and behaviours expected by the dental profession and the public. It is established and nurtured through adherence to the principles of ethical reasoning: respect for autonomy, beneficence, non-maleficence, and social justice.’

Professionalism is a core foundation to the practice of health professionals, however, Passi and colleagues (2010) identified that there are currently no guidelines how to best develop professionalism competency among medical students. This may perhaps include other health professional education as well, including dentistry.

Shapiro and colleagues (2015) identified that there are at least two significant issues regarding professionalism in medical education: 1) the content of professionalism itself, and how it is defined, and 2) how professionalism is measured and achieved. Although some approaches have been shown to have positive outcomes, most approaches to teaching professionalism, which are implicitly or explicitly rooted competency models, have not documented significant success ( Shapiro et al, 2015).

A student portfolio is a common component of summative assessment used in clinical education, and are used within the OHT program at the Melbourne Dental School. Portfolios have been criticised to be time consuming, have had inadequate support and mentoring despite some evidence of positive impact ( Driessen et al, 2007). Furthermore, the quality of self-reflection cannot be assumed, the time commitment can be a deterrent, and this could detract from other learning opportunities unless mandated by the demands of assessment ( Buckley et al, 2009). However, student portfolios it still viewed to have an important role in documenting evidence on performance and assessment ( Wilkinson et al, 2009).

In 2015, notifications, imposed sanctions, and in some cases de-registration against dental practitioners for reported incidences of breaches in infection control in New South Wales, Australia, have refreshed the essential need for dental practitioners to promote public safety and protection. It is a reminder for prudence in professionalism. There is evidence that unprofessional behaviour as students may predict subsequent poor performance in independent practice ( Papadakis et al, 2005). Corrective actions towards unprofessional behaviour and promoting professionalism is critical to preserve public confidence.

Zijlstra-Shaw and colleagues (2012) published a paper titled: ‘Assessing professionalism within dental education; the need for a definition’. Whilst highly relevant, the paper is broadly descriptive in nature and written as a narrative review, which did not have elements of a systematic review methodology. Passi and colleagues (2010) published a systematic review with the primary objective to review the evidence within the medical profession. To date, there were no published papers that focused on developing professionalism in dentistry.

## Methods

**Research Aim:** To investigate: “What is the evidence for clinical education practice approaches to develop professionalism in dentistry?”

### Research Questions:

1. What different types of clinical education practice approaches exist to promote professionalism in dentistry?
2. What is the quality and strength of evidence to support these clinical education practice approaches?

**Philosophical Assumptions:** The epistemological assumption for this research is of the post-positivist. It included quantitative, qualitative and mixed methods research papers. Through the post-positivist lens, data is collected from multiple sources to reach a consensus to make informed conclusions about social phenomena. The theory underpinning post-positivist research is to value problem-setting rather than problem-solving ( Ryan, 2006). With this approach, it is critical to recognise the conclusions made are influenced by the interpretation of the researchers and the research context. Any research outcomes is subjected to the researchers’ prejudice. Thus, this review is intended to provide a coherent body of work to interested readers.

**Methodology:** This research was conducted using a non-Cochrane/non-Campbell systematic literature review. Ethics approval was not required and followed the principles of the Declaration of Helsinki.

A search of the following electronic databases and journals was undertaken: Medline; EMBASE, CINAHL, Web of Science, and Discovery within the Journal of Dental Education, European Journal of Dental Education, and the Journal of Dentistry, Oral Medicine and Dental Education from 2000 to June 2016.

The focus of the literature search was for the following disciplines within dentistry: dental hygiene, dental therapy, oral health therapy, and dentistry. The search used natural language key words: “education”; “student”; “dental” or “oral health” or dentistry”; “professionalism” or “ethic”.

An Endnote X7 Reference Manager (Clarivate Analytics) library was created to catalogue these references. The bibliography created from the search produced a list of references with title and/or abstract. Duplicate entries were manually removed.

The database of potential papers was imported into ‘Covidence’, and screened for further assessment according to the title and/or abstract by TMN. Full-text articles were obtained, and the eligibility of each potential study with full-text screening was assessed independently by two investigators (TMN and KLN) based on the inclusion criteria ( Table 1), and then jointly to reach an agreed list. Quality appraisal, strength of evidence, and educational outcome evaluation were independently assessed by both investigators. Conflicts were discussed to arrive with a consensus score.

**Table 1.** The systematic review selection criteria.

Search Parameters	Inclusion Criteria	Exclusion Criteria
Clinical Education	Clinical setting Preclinical setting	Non-clinical setting
Dental Hygiene / Dental Therapy / Oral Health Therapy / Dentistry	Undergraduate programs Postgraduate programs	Registrable dental practitioners
Design	Systematic review Literature review Original empirical research(any design)	Letters Editorial Commentary
Language	English	Non-English
Publication Date	Papers published from 2000 to July 2016	Papers published before 2000
Articles	Full-text Peer reviewed	Abstract Non-peer reviewed

**Quality Appraisal:** Papers that meet the selection criteria were critically assessed against a quality appraisal framework. The quality of papers were assessed against the following questions ( Kuper et al, 2008):

1. Was the sample used in the study appropriate to its research question?
2. Were the data collected appropriately?
3. Were the data analysed appropriately?
4. Can I transfer the results of this study to my own setting?
5. Does the study adequately address potential ethical issues, including reflexivity?
6. Overall: is what the researchers did clear?

**Strength of Evidence:** Potential papers were scored on a scale of 1 - 5 for the strengths of the findings ( Colthart et al, 2008):

Grade 1: No clear conclusions can be drawn. Not significant.

Grade 2: Results ambiguous, but there appears to be a trend.

Grade 3: Conclusions can probably be based on the results.

Grade 4: Results are clear and very likely to be true.

Grade 5: Results are unequivocal.

**Educational Outcomes:** The educational outcomes were based on Kirkpatrick Hierarchy ( Table 1), adapted by Steinert and colleagues (2006) ( Table 2).

**Table 2.** Evaluating educational outcomes based on Kirkpatrick’s model.

**Level 1 REACTION** Participants’ views on the learning experience, its organisation, presentation, content, teaching methods and quality of instruction.
**Level 2A LEARNING: Change in attitudes** Changes in the attitudes or perceptions among participant groups towards teaching and learning.
**Level 2B LEARNING: Modification of knowledge or skills** For knowledge, this relates to the acquisition of concepts, procedures and principles; for skills, this relates to the acquisition of thinking/problem-solving, psychomotor and social skills.
**Level 3 BEHAVIOUR: Change in behaviours** Documents the transfer of learning to the workplace or willingness of learners to apply new knowledge and skills.
**Level 4A RESULTS: Change in the system/organisational practice** Refers to wider changes in the organisation, attributable to the educational program.
**Level 4B RESULTS: Change among the participants-students, residents or colleagues** Refers to improvement in student or resident learning/performance as a direct result of the educational intervention.

**Data Synthesis:** The textual narrative synthesis research method was used. It is an approach that ranks studies into homogenous groups ( Barnett-Page and Thomas, 2009). This approach offered an appropriate mechanism for ensuring a focus on the research questions. Relevant data extraction from the included papers were managed using nVivo 11 (QSR International Pty Ltd) by TMN.

### Results

The literature search yielded a total of 311 articles ( Appendix 1). A further 115 articles were removed as a result of duplicate entries. Of the total of 195 articles subjected to title and/or abstract screening, 34 full-text papers were reviewed against the inclusion criteria. Of these papers, 15 papers met the inclusion criteria as shown on the PRISMA flow diagram ( Figure 1)(Moher et al, 2009). No papers were related to developing professionalism at a post-registration qualification level for dental specialist course programs.

**Figure:**
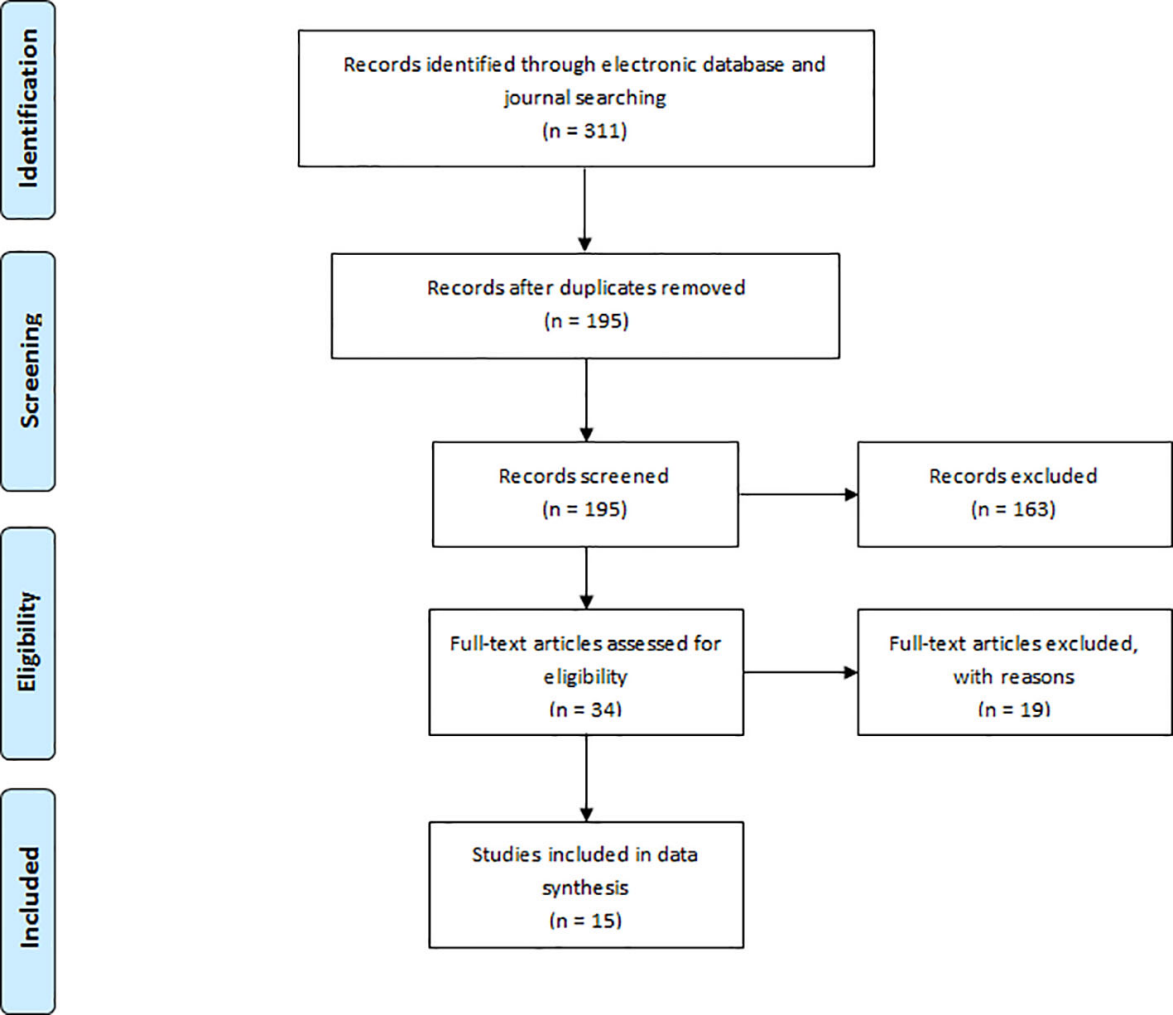


**Focus of Included Papers:** There were three main themes supporting the development of professionalism within dentistry: Curriculum Design, Teaching and Learning Methods, and Assessment. This categorisation was adopted from Passi and colleagues (2010).

The various approaches for clinical education to promote professionalism in dentistry were:

• Faculty training
• ‘Holistic’ clinical education
• Incident reporting system
• Numerical professional judgment grades
• Reflective written approaches
• Rotational outreach community clinical practice placements
• Standardised patient clinical encounters
• The use of videos

**Table 3.**
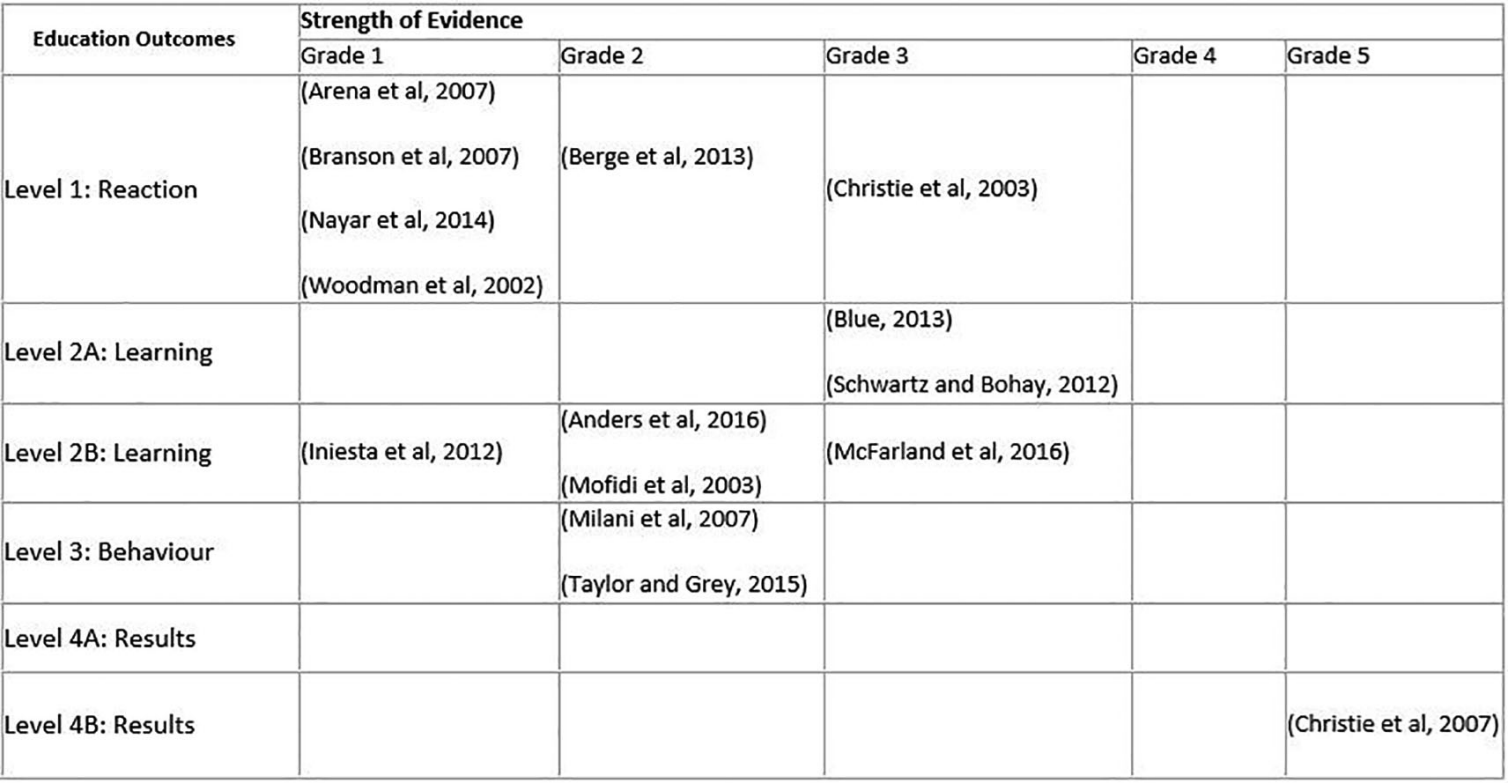
Summary results for the education outcomes and strength of evidence.

### Curriculum Design

A total of five papers were related to curriculum design. Four articles reported the student’s educational outcomes on learning regarding professionalism and other student competencies ( Arena et al, 2007, Berge et al, 2013, Nayar et al, 2014, McFarland et al, 2016). One article was in relation to dental hygiene students ( Blue, 2013).

**Level 1 - Reaction:** A clinical education program was introduced into the curriculum for dental students in 2002 at the University of Western Australia consisting of forty weeks of blended learning environments at community-based, hospital-based, and some private dental practices ( Arena et al, 2007). The program was extended from a four-year to a five-year degree, the 5th year being primarily of clinical education practice. The perception of all dental graduates in the 2000, 2001 and 2004 were assessed based on a survey questionnaire instrument developed by medical educators from the university. For all three thematic clusters on ‘General Skills’, ‘General Clinical Skills’ and ‘Specific Clinical Skills’, there was no statistically significant differences found between those students undertaking the new program and those with the previous curriculum ( Arena et al, 2007). Although professionalism was not the primary outcome of interest, some of the learning outcomes were related to professionalism.

The clinical educator perspective on achieving competencies of the American Dental Education Association (ADEA) for the New General Dentist was evaluated at the University of Nebraska Medical Center ( Nayar et al, 2014). Dental students begin clinical practice rotations from 3rd year before completion of studies in 4th (final) year. They are required to complete 2 two-week community-based clinical education practice in a rural dental private practice and in a public health dental clinic. The clinical educators reported ‘Excellent’ or ‘Very good’ for dental students meeting most of the ADEA domains. In the ‘Practice Management and Informatics’ domain, there was a skew for the perception to be ‘Very good’ or ‘Good’; For the domain of professionalism, 86% of respondents felt the program effectiveness was ‘Excellent’ or ‘Very good’ ( Nayar et al, 2014). No statistical analysis was conducted for the outcomes between the cohort responses in 2012 and 2013.

Graduating dental student perspectives were evaluated on the new curriculum introduced from 2003 at the University of Bergen, Norway ( Berge et al, 2013). Comparisons were made with the 2000 student cohort who studied with the previous curriculum. ‘Holistic teaching’ were adopted to align dental education to be patient-centred and student-centred. Student clinical practice was introduced earlier in the curriculum, and the teaching of dental specialties was integrated into general practice. In addition, the scoring system was replaced with a pass/fail grade for both clinical courses and theoretical exams. A statistical significant difference was found for dental students in the 2003 cohort who expressed that there were ‘Too few’ or ‘Much too few’ available clinical instructors during clinical education sessions, and students were less satisfied with the feedback. More than 50% and 75% of dental students in both groups expressed the opinion that clinical educators were ‘Poorly’ or ‘Very poorly’ calibrated, and dissatisfied with the feedback. Dental students were indifferent to the preferred dichotomous (pass/not pass) and a graded (1-12, >6: passed) scale.

**Level 2A - Learning:** Blue (2013) reported curriculum redesign in the 2008-2009 academic year at the dental hygiene program at the University of Minnesota. Students were required to engage in providing clinical care in urban and rural community settings. Guided reflections tasks were provided to help students reflect on the social issues of the community and consolidating what was learnt in the classroom. This approach was complemented with the new curriculum on professionalism consisting of written assignments. Student post-evaluation educational outcomes were assessed using a five-point Likert scale, and *The Attitudes Toward Health Care* instrument, in 2009, 2010 and 2011. The increased in mean attitude scores were statistically significantly different for the domains of ‘Societal Expectations’, ‘Dentist/Student Responsibility’ and Personal Efficacy’; the ‘Access to Care’ domain was not significantly different ( Blue, 2013).

**Level 2B - Learning:** The dental student perspective on meeting the ADEA competencies of the program ( Nayar et al, 2014) was reported by McFarland and colleagues (2016). A retrospective post-survey questionnaire was completed by the 2011/12 and 2012/13 4th (final) year dental student cohorts. These surveys asked participants to rate their program effectiveness in relation to improving the students’ competence in the six ADEA competency domains. For both year cohorts, there was statically significant mean difference for all domains in favour for the outreach clinical practice placements, including for the ‘Professionalism’ domain, (p<0.01) ( McFarland et al, 2016).

**Level 3 - Behaviour:** No studies were found in this category.

**Level 4A - Results:** No studies were found in this category.

**Level 4B - Results:** No studies were found in this category.

### Teaching and Learning

Five articles reported the use or were related to different methods for teaching and learning. Two papers reported approaches on teaching in a preclinical environment (Anders et al, 2016, Schwartz and Bohay, 2012) and three papers were related to developing reflective practice through the use of written methods ( Woodman et al, 2002, Mofidi et al, 2003, Branson et al, 2007).

**Level 1 - Reaction:** Dental hygiene students were required to maintain a journal when providing clinical care over two weeks at Federally Qualified Health Centers throughout Missouri ( Branson et al, 2007). Quantitative measures reported the type and number of procedures the students performed. The most common three themes the students wrote about were: ‘Standard of Care Rendered’, ‘The Level of Difficulty Treating the Oral Conditions’, and ‘Treatment Techniques in Practice vs. School’. A post-survey questionnaire revealed positive responses from the perspective of the student, the dental clinical and facility staff ( Branson et al, 2007); no statistical analysis was performed.

Woodman and colleagues (2002) described their approach through a structured documentation to demonstrate dental students’ and dental therapy students’ reflective practice from their clinical experiences. The dental students were 3rd year undergraduates, and the dental therapy students were in their 1st year but most had clinical experiences as a dental nurse or a dental hygienist. Through a qualitative thematic content analysis, there were no marked differences in the responses between the two student disciplines. The types of incidents the students reflected on were regarding the mistakes that they made, patient management difficulties, ‘milestones’ of their dental training, and technical aspects of dental treatment. Negative feelings were also reported about themselves, and ‘systems’ factors such as the clinical educators and school administration.

**Level 2A - Learning:** Schwartz and Bohay (2012) used patient videos from volunteers who shared their experiences involving insensitivities when visiting the dentist, dental treatment errors, or issues regarding dental access to care. Students were required to write a 1,000-word reflective journal. Prior to the viewing of the patient videos, over 80% of students felt they understood the meaning of ‘empathy’ and how ‘empathetic’ they are to their patients. For the post-survey, dental students in 2nd year cohort felt the video significantly increased their likelihood of participating in government-sponsored dental plans (88%), enhancing their commitment to professionalism (84%), affected the level of compassion for patients (94%), and the reflective journal raised the level of empathy for patients (72%) compared to the 3rd (final) year cohort (78%, 67%, 76%, 44%, respectively). There were statistically significant mean difference that 3rd year cohort had a lower mean score of 110.28 based on the Jefferson Scale of Empathy compared to the 2nd year cohort, with a mean score of 117.13 ( Schwartz and Bohay, 2012).

**Level 2B - Learning:** One study reported an interprofessional practice simulation through the use of standardised patients in hospital setting at the University at Buffalo (Anders et al, 2016). The doctor of nursing practice student was required to assess the patient, and perform a clinical handover to the visiting ‘consultant’ 4th (final) year dental student. Both students then collaborated together to discuss the findings with the patient and discuss the management plan. The simulation was observed remotely by faculty staff, and recorded for later viewing. A competency standardised patient checklist ranged between 67% and 100%. The dental student self-evaluation checklist ranged between 60% and 88%. There was a strong correlation between the standardised patient checklist and the student self-evaluation checklist only in the area of ‘Communication’, with Pearson correlation of 0.79. For the Interprofessional Competence Scale, an instrument developed by the faculty staff, there was a mean score of 5.8, indicating positive outcomes in areas of ‘Values/ethics’, ‘Roles/responsibilities’, ‘Interprofessional communication’ and ‘Teams/teamwork’ (Anders et al, 2016).

Mofidi and colleagues (2003) asked final year dental students to write a reflection essay about a critical incident that occurred during their clinical education rotation regarding professional and personal issues. The critical incidents were categorised into three broad themes: ‘Personal and Professional Growth’, ‘Enhanced Awareness’, and ‘Commitment to Service’. Two of the most common subthemes were ‘Caring’ (65%) and ‘Complexity of patients’ lives’ (66%), and the least two common subthemes were ‘Self-confidence’ (20%) and ‘Making a difference’ (20%); more than half of the students shared about their critical incidents on communication ( Mofidi et al, 2003).

**Level 3 - Behaviour:** No studies were found in this category.

**Level 4A - Results:** No studies were found in this category.

**Level 4B - Results:** No studies were found in this category.

### Assessment

Three papers were student-focused ( Christie et al, 2003, Iniesta et al, 2012, Taylor and Grey, 2015), and two studies were teacher-focused in relation to assessment ( Christie et al, 2007, Milani et al, 2007).

**Level 1 - Reaction:** Christie and colleagues (2003) identified the need evaluate the curriculum content and evaluation methods for assessing ethical reasoning and professionalism with the dental hygiene program at Idaho State University. The first phase involved developing competencies of ethics and professionalism by faculty staff. They were reviewed against the curriculum content for all subjects within the program through a survey questionnaire and individual faculty staff interviews. A new ethics and professionalism competency document was created incorporating those feedback mechanisms. Core values for each of the five related competencies were assigned based upon the American Dental Hygienists’ Association *Code of Ethics for Dental Hygienists.* Students’ clinical performance was evaluated using professional judgment grades issued by clinical faculty members for each patient, and are encouraged to write a comment related to the grades issued. The study found that there were significant differences in favour of senior dental hygiene students’ attitudes compared to junior dental hygiene students regarding ( Christie et al, 2003):

• Their feeling familiar enough to apply laws and regulations governing dental hygiene in patient care
• Their perceived ability to record all required components of patient care in the record of services
• The importance they placed on informing their supervisor/instructor when making a mistake that could potentially affect others
• Their belief in the fundamental importance of community service activity
• Their perceived honesty and openness with peers
• The value placed on being consistently cognisant of ergonomics

The remaining 28 survey items showed no significant difference about ethics and professionalism in dental hygiene practice ( Christie et al, 2003).

**Level 2A - Learning:** No studies were found in this category.

**Level 2B - Learning:** Iniesta and colleagues (2012) reported the implementation of a modified mini-Clinical Evaluation Exercise (mini-CEX). It involved the direct observation of clinical skills for dental students. Groups of four students were assigned to a single clinical educator throughout a 2-month period. The maximum time allowed for each session was three hours, and the clinical educator provided subjective assessment based on a 10-point scale, and feedback provided during the clinical education session. For all domains of clinical skills assessed, the mean scores improved in the second month compared to the first month. The greatest improvement was in ergonomics and posture, and the least improvement was in manual skills; the mean score change in the domain of professionalism was 5.74 to 6.26 ( Iniesta et al, 2012).

**Level 3 - Behaviour:** Dental students at the University of the Pacific were assessed for each dental speciality by all faculty staff ( Milani et al, 2007). The numerical competency rating system was used to evaluate clinical judgment, patient management, and skills. Clinical educators were also encouraged to record comments, which are transcribed by the student and reviewed by Student Academic Performance and Promotion Committee to assist student learning where appropriate. Approximate half of the comments were negative and positive in nature; of the total number of comments, 37% were focused on clinical performance, 43% on patient interactions, and 20% were related to relationships with faculty staff or the dental clinic system ( Milani et al, 2007). In addition, it was reported that 12% of the comments was related to professionalism, of which 70% of these comments were negative.

A critical incident reporting system was introduced within the dental student curriculum at the University of Manchester ( Taylor and Grey, 2015). The authors recognised that professionalism incidences were occurring external to the formal teaching sessions, both positive and negative behaviours. A three-card report categories were created: ‘red’ for serious unprofessional behaviour involving dishonesty or endangering patient safety, ‘yellow’ for minor unprofessional behaviours and ‘green’ for positive professional behaviours. It can be given by any member of staff such as administrative, dental nursing, clinical and academic, and in any environment. The professionalism incidents are considered as part of the eligibility criteria to sit examinations. Students with three yellow cards and one red card must meet with their faculty staff head of year with potential further action. The student with the most green cards at graduation would receive a prize. Faculty staff received training utilising the professionalism critical incident system. In the 2 years since implementation, the majority of cards were awarded by administrative (29%) and part-time clinical staff (24%); 76% of cards were awarded in clinical environments (clinic and pre-clinical) with 24% relating to non-clinical environments ( Taylor and Grey, 2015). For unprofessional behaviours, the most commonly reported incident was in regards to a lack of conscientiousness. There was a significant increase of green card reports submitted in the second year of implementation.

**Level 4A - Results:** No studies were found in this category.

**Level 4B - Results:** The authentic evaluation processes was addressed in the dental hygiene by trying to increase the number of written comments to support professional judgement grades ( Christie et al, 2007). Evaluated over a three-year period, all faculty staff responsible for clinical teaching was invited to a 4-hour faculty development workshop. Laminated cards were given to clinical educators listing the core values and alternative descriptors to support the evaluation of students’ performance regarding professionalism in the clinical setting. As a result, clinical educators provided a greater proportion of comments related to the core values of professionalism prior to the workshop for all student year cohorts. Multiple sources of mixed methods evaluation data support positive trends that the faculty development program promoted professionalism for dental hygiene students at Idaho State University ( Christie et al, 2007).

## Discussion

The range of strategies to promote professionalism in the clinical education context in dentistry are explored in more detail.

**Rotational outreach community clinical practice placements:** Arena and colleagues (2007) documented the educational outcomes on the implementation of year-long rotational clinical practice placements. The strength of the evidence was Grade 1. Four papers reported the educational outcomes on professionalism for rotational outreach community clinical practice placements, which were not year-long. Two papers had strength of evidence Grade 3 ( Blue, 2013, McFarland et al, 2016), while the other paper was Grade 1 ( Nayar et al, 2014). Overall, there is evidence to demonstrate the benefits of outreach clinical placements for the development of dental professionalism. It would seem reasonable that this approach is beneficial because it simulates and tests clinical skills and behaviours in an authentic way using workplace-based clinical practice.

**Numerical professional judgement grades:** Four papers reported the use of numerical professional judgement grades as part of the clinical education practice, where comments are encouraged to support the assessment ( Christie et al, 2003, Christie et al, 2007, Milani et al, 2007, Iniesta et al, 2012). However, this is largely subject to individual clinical educator perceptions, and questions remain unanswered regarding intra-rater (individual) and inter-rater (colleagues) reliability and validity. Satisfactory inter-rater reliability is achievable as reported in a study of two educators assessing undergraduate physiotherapist clinical performance (Meldrum et al, 2008).

Numerical grades are useful to determine how well students perform. This is particularly required when there are high stakes in deciding the students’ performance for the course subject grade, which can make a difference between ‘high distinction’ and ‘distinction’ (which may be critical for entry into postgraduate course programs) or ‘pass’ and ‘fail’. Two papers reported the use of numerical professional judgements grades for overall performance in professionalism for each clinical encounter ( Christie et al, 2007, Christie et al, 2003), while student clinical performance was assessed within various competency domains for each clinical encounter ( Iniesta et al, 2012) or were quarterly assessed three-monthly ( Milani et al, 2007).

The strength of evidence for the utilisation of numerical professional judgement grades included Grade 1 ( Iniesta et al, 2012) and Grade 3 ( Christie et al, 2003, Milani et al, 2007). It should be noted that both studies reported by Iniesta and colleagues (2012) and Christie and colleagues (2003) reported clinical educator training, which may influence the their results. Our literature review suggests that numerical professional judgement grades are likely to be beneficial.

**Reflective written approaches:** Four papers reported different types of reflective written approaches in promote professionalism in dentistry ( Blue, 2013, Branson et al, 2007, Mofidi et al, 2003, Woodman et al, 2002). The strength of evidence varied from Grade 1 ( Branson et al, 2007, Woodman et al, 2002), Grade 2 ( Mofidi et al, 2003) and Grade 3 ( Blue, 2013). A study evaluating the usefulness of reflective writing as part of the student portfolio to improve clinical performance statistically determined that it required 14 writing samples per student to make meaningful conclusions about reflective capacity ( Moniz et al, 2015). Earlier work in psychiatry using the student portfolio demonstrated scores improved with years of training and were correlated with knowledge but not for clinical performance ( O’sullivan et al, 2005). Reflective written approaches through service-learning, where students undertake a student project to implement an oral health education/promotion activity within a target priority population, have reported educational outcomes towards social justice ( Behar-Horenstein et al, 2015, Brondani, 2010, Brondani, 2012, Dharamsi et al, 2010, Keselyak et al, 2007). It may be beneficial to review and modify how the student portfolios are used in a consolidated constructive way to develop professionalism.

**The use of videos:** Schwartz and Bohay (2012) used videos in a preclinical setting as a way to develop professionalism and empathy. It would seem appropriate, useful and relevant that the use of videos to show how registered dental practitioners communicate and interact with their patients would be of value for students in developing professionalism. The strength of evidence is Grade 3. Our research found that there is a lack of triangulated evidence to support the use of videos to promote dental professionalism.

**Standardised patient clinical encounters:** The use of standardised patient clinical encounters is widely used in medical education. One study reported by Anders and colleagues (2016) has a strength of evidence Grade 2. Standardised patients require investment to train actors, can be labour intensive, and do not generate clinical productivity compared to direct provision of patient care. Furthermore, the research reported was of an interdisciplinary activity involving dental students and doctor of nursing practice students, and had a cost of US$100 per student per scenario (Anders et al., 2016). The scenario piloted is not typical of routine clinical practice for dental practitioners unless they work in acute care hospital settings.

**‘Holistic’ clinical education:** The ‘holistic’ teaching approach undertaken by Berge and colleagues (2013) had strength of evidence Grade 1. This may be generally due to the research and evaluation methodology rather than the approach itself, as it the fundamental principles has merit from the educational and public expectation point of view. In real life situations, the provision of dental services is largely not performed isolated dental speciality ‘silos’, rather comprehensive care is provided based on a thorough clinical risk assessment of the patient. The only exception would be in circumstances where a patient is seeking emergency dental care only, or when patients are referred to a dental specialist.

**Incident reporting system:** The work undertaken by Taylor and Grey (2015) in the implementation and evaluation of the incident reporting system has some merit in developing professionalism. It required the training and calibration of faculty staff and non-university employees involved in the clinical education practice of dental students to appropriately use this assessment approach. It was the only study to be inclusive from the perspective of other support teaching staff. The assessment of professionalism confined to solely in the clinical education environment may not be sufficient to capture professional behaviours that may occur external to the formal curriculum. This is in reflection that professionalism is a long-term demonstration of competency behaviours, and requires a ‘whole being’ transformation in how individuals should be behaving as a dental practitioner. The incident reporting system had strength of evidence Grade 2.

**Faculty training:** There was good evidence reported by Christie and colleagues (2007) that documented higher levels of educational outcomes as a result of faculty training to promote dental professionalism. Although the research did not capture student development in professionalism, the study objective was to encourage clinical educators to increase the number of comments to support the numerical professional judgement grades of the clinical practice encounter. The strength of the evidence was Grade 5, with educational outcomes of Level 4B: Results. Whilst it is difficult to make a definitive conclusion that faculty training is beneficial based on one this study, other studies has faculty training ( Berge et al, 2013, Iniesta et al, 2012, Taylor and Grey, 2015). More than 50% of dental students felt that the clinical educators were poorly or very poorly calibrated ( Berge et al, 2013), while another paper not included in this review identified that inconsistent and sometimes insensitive (patronising, rude) feedback from faculty was one of four major themes of dental student clinical education experiences ( Henzi et al, 2006). Since most faculty members undergo adhoc training in the absence of formal education training, where they hope to enhance skills after they assume their teaching roles ( Srinivasan et al, 2011), it is important to consider institutional led-faculty training opportunities.

The establishment for the standard how professionalism is defined amongst colleagues is important to assure the reliability and the validity of assessment on student performance. The need for faculty training has gained increasing scrutiny as demonstrated in the work on faculty development for learning and teaching of medical professionalism in Saudi Arabia ( Al-Eraky et al, 2015). Alongside faculty training to enhance the development of student professionalism is the potential effect for student evaluating clinical educators’ performance to help drive their learning needs ( Schaub-de Jong et al, 2011, Todhunter et al, 2011, Young et al, 2014). The utilisation of faculty training appears to have merit to promote professionalism in dentistry.

**Study Strengths:** Our research had several strengths. Firstly, we systematically reviewed the literature with regards to the available evidence to inform how we can develop dental professionalism. It was conducted consistent with best practice medical education research methodology. The study population search terms consisted of a broad range of dental practitioners. Included papers were subjected to quality appraisal, strength of evidence and educational outcomes grading by two reviewers. This adds research rigour to the research methodology to generate valid results. We can comfortably say the research findings are generalisable to the dental profession.

**Limitations:** Whilst there a several qualities to support the strength of our research, there are certainly areas of improvement. Not all eligible studies would be identified since the search methodology did not source the grey literature or other databases, it is influenced by publication bias, and the search terms was ‘professionalism’ and ‘ethics’ only with no variations of the term, or key related terms. Furthermore, the rigour of our research methodology should incorporate more reviewers, who should be involved the research design including independent reviewing of the title/abstract, full-text review, quality appraisal, strength of evidence and educational outcomes grading. Bearman and colleagues (2012) recommended that a systematic review should:

• expand the systematic search to unpublished reports to avoid publication bias
• having a research team that covers national boundaries
• follow a research protocol *a priori* and peer reviewed
• paper selection, data extraction and quality assessment is performed independently by at least two reviewers
• be subjected to peer review and editorial review by the relevant best-practice systematic literature review group/network

A critical component of reporting, summarising and making conclusions when undertaking research is to impart reflexivity for the readership. We identified all included papers had major research methodology flaw: dental professionalism was not defined. This is acknowledged also in the work by Zijlstra-Shaw and colleagues (2012) that if a clear definition of professionalism as a construct is not proposed, the authenticity and validity for assessing professionalism is undermined. Few studies in our review evaluated student attitudes towards the principle of social justice, such as their intent to work in publicly government funded agencies ( Blue, 2013, Christie et al, 2003, Mofidi et al, 2003, Schwartz and Bohay, 2012).

In general, there were limited good quality papers that evaluated dental professionalism as a primary outcome, and most papers were of related to low levels of educational outcomes. Most papers did not state their philosophical assumption or research theory to support and justify their research approach. Many evaluation measures reported did not clearly demonstrate reliability or validity, and few papers reported pilot studies (Anders et al, 2016, Iniesta et al, 2012).

## Conclusions

In summary, eight different types of clinical education practice approaches to promote dental professionalism was identified and explored in this systematic review. Most reported methods of clinical education were of low quality and focused on low level educational outcomes, which is common finding from other health professional disciplines. Future high quality research needs to clearly define professionalism in dentistry. There are opportunities to have an inter-professional approach to develop and assess professionalism.

## Take Home Messages

• A working definition of dental professionalism still needs to be developed
• Eight different clinical education approaches were identified to promote dental professionalism
• Most papers were of poor quality and were related to low level educational outcomes
• The findings is consistent with the evidence across other health professions internationally
• There is an opportunity for an inter-professional approach to develop and assess professionalism

## Notes On Contributors

Mr Tan Minh Nguyen developed the research protocol, study design, data collection and analysis, interpretation of the findings and preparation of the manuscript.

Dr Derek Jones was involved with the research protocol, study design, and review of the manuscript.

Mr Kinh Luan Ngo assisted with the data analysis and review of the manuscript.

Dr Melanie Jane Hayes assisted with the research protocol, study design, and review of the manuscript.

## Appendices

### Appendix 1

**Table 5.** Systematic literature review search results.

Electronic Database / Journal	Search Syntax	Search Results
Medline	1. education.ab.	26,2499
	2. (“dental” or “oral health” or “dentistry”).ab.	12,4691
	3. student.ab.	48,677
	4. (professionalism or ethic*).ab.	67,632
	5. 1 and 2 and 3 and 4	44
	6. 5 and 2000:2016.(sa_year).	43
EMBASE	1. education.ab.	35,9970
	2. (“dental” or “oral health” or “dentistry”).ab.	13,7333
	3. student.ab.	67,906
	4. (professionalism or ethic*).ab.	100,336
	5. 1 and 2 and 3 and 4	47
	6. 5 and 2000:2016.(sa_year).	46
CINAHL	S1. AB education	95,060
	S2. AB dental OR AB oral health ORAB dentistry	99,650
	S3. AB student	57,570
	S4. AB professionalism OR AB ethic*	19,149
	S5. S1 AND S2 AND S3 AND S4 (Limiters - Published Date: 2000-2016)	10
Web of Science	#1. TOPIC: (education) (Timespan=2000-2016)	460,654
	#2. TOPIC: (dental) OR TOPIC: (“oral health”) OR TOPIC: (“dentistry”)(Timespan=2000-2016)	103,087
	#3. TOPIC: (student)(Timespan=2000-2016)	373,009
	#4. TOPIC: (professionalism) OR TOPIC: (ethic*) (Timespan=2000-2016)	124,669
	#5. #4 AND #3 AND #2 AND #1 (Timespan=2000-2016)	150
Discovery	S1 SO “journal of dental education” OR SO “european journal of dental education” OR SO ( “journal of dentistry, oral medicine and dental education”)	26,677
	S2. AB education	15,350,310
	S3. AB dental OR AB “oral health” OR AB “dentistry”	1,451,705
	S4. AB student	19,116,945
	S5. AB professionalism OR AB ethic*	2,133,837
	S6. S1 AND S2 AND S3 AND S4 AND S5 (Limiters - Published Date: 2000-2016)	174
