# Supplementary material for: Developing Professionalism in Dentistry: A Systematic Review
Source: MedEdPublish (2016). 2017 May 17;6:85. [Version 1] doi: 10.15694/mep.2017.000085 (PMC10885255; doi:10.15694/mep.2017.000085)
Supplement: Peer review history

# Reviewer report: Reviewer response for version 1

**DOI:** 10.21956/mep.19278.r28039

**Published:** 2017-05-24

**Authors:** Sandra Zijlstra-Shaw

**Affiliations:** 1 School of Clinical Dentistry,

This review has been migrated. The reviewer awarded 3 stars out of 5 I concur with the previous reviewer that this is a very interesting paper, however maybe not for the same reasons. Unfortunately I am concerned that this is not a well performed review and am at least as concerned as the first reviewer about the “lack” of evidence. The authors suggest that they carried out a scoping review looking for definitions of professionalism in dentistry and could find none. However, I am aware of at least 3 papers which do provide a definition (Welie 2004, Trathen and Gallagher 2009 and my own Zijlstra-Shaw et al 2013). The other issue I have is with the search terms used in the systematic review. Whilst I am conscious that the authors recognise the limitations of their search terms, I am not surprised that they struggled to find literature referring to professional behaviours in most of their categories, as they didn’t search specifically for this. Furthermore in the first decade of 2000 much of the discussion in the literature was around the nomenclature and whether professionalism was professional behaviours or professional attitudes or some combination of the two. This has severely limited the quality of the paper in my eyes as, once again, I am aware of missed papers.All this having been said, I applaud the effort made to carry out a systematic review in this important area and agree with the conclusion that more, higher quality research is needed, especially with respect to the reliability and validity of the assessment methods used. Following their cut-off date there has been at least one addition to the dental education literature which has a clear definition and demonstrates both validity and reliability. I am also aware that there is ongoing research in this area so a repeat of this review using broader search terms and incorporating more reviewers, as suggested by the authors would be a worthwhile exercise.

Reviewer Expertise:

NA

No decision status is available

# Reviewer report: Reviewer response for version 1

**DOI:** 10.21956/mep.19278.r28038

**Published:** 2017-05-17

**Authors:** Trevor Gibbs

**Affiliations:** 1 AMEE

This review has been migrated. The reviewer awarded 5 stars out of 5 A very interesting paper that I enjoyed reading and learned from; made all the more relevant by my reading of it just after I have returned from my quarterly visit to the Dental Therapist ! - perhaps I should have read it earlier and then I could have asked my therapist about her training, although I had no trouble recognising the high level of professionalism shown.Joking aside, I do believe that this is a well performed review which probably gave the authors confirmation of the results they were expecting. However, further research and subsequent development is build upon such reviews and so I would recommend this paper to all those involved in the curricula design of professionalism, specifically those training the non-medical professionals. I may also add that the references are particularly important to those new to the subject and are a useful point of reference.

Reviewer Expertise:

NA

No decision status is available

# Reviewer report: Reviewer response for version 1

**DOI:** 10.21956/mep.19278.r28037

**Published:** 2017-05-17

**Authors:** Richard Hays

**Affiliations:** 1 James Cook University

This review has been migrated. The reviewer awarded 5 stars out of 5 This is a well researched paper on professionalism in dentistry. I particularly like the application to Kirkpatrick's hierarchy, consideration of teaching and assessment, and then the strategies that might enhance professionalism. I do wonder at the 'lack' of evidence, because I think that many health professions have much in common. Much more is written about professionalism in medicine, so for me the focus should be on any differences between professionalism in medicine/other professions and dentistry. In medical education it is fair to say that formal teaching in professionalism is perhaps the least important strategy, whereas role modelling, incorporation into clinical practice and workplace-based assessment may be the most important. This is a useful paper for all health professions educators to read.

Reviewer Expertise:

NA

No decision status is available
